# Impact of protective haemoglobins C and S on *P. falciparum* malaria transmission in endemic area

**DOI:** 10.1186/1475-2875-9-S2-O17

**Published:** 2010-10-20

**Authors:** Louis C Gouagna, Germana Bancone, Frank Yao, Carlo Costantini, Jean-Bosco Ouedraogo, David Modiano

**Affiliations:** 1 Institut de Recherche pour le Développement(IRD), Unité de Recherche 16, Montpellier, France; 2 Centre de Recherche et de Veille sur les maladies Emergentes dans l'Océan Indien (CRVOI), Sainte Clotilde La Réunion; 3 Department of Public Health Sciences, University of Rome 'La Sapienza', Rome, Italy; 4 Institut de Récherche en Sciences de la Santé (IRSS), Direction Régionale de Bobo-Dioulasso, Bobo Dioulasso, Burkina Faso; 5 IRD, Unité de Recherche 016, Organisation de Coordination pour la lutte contre les Endémies en Afrique Centrale, Yaoundé, Cameroon

## 

Human genetic factors play a key role in determining the resistance/ susceptibility to infectious diseases. It is unknown whether genetic makeup may also influence host efficiency to transmit pathogens. With regard to malaria, a major selective force in recent human evolution, protective erythrocyte variants have been describe, but little is known as to their possible impact on the transmission of the parasite from the human host to the *Anopheles* vector.

Here, we performed genetic, parasitological and entomological investigations involving a total of 3799 human subjects carrying the HbAA, HbAS, HbAC and HbCC β-globin genotypes in order to determine whether variation in host infectivity to the *Anopheles* vector can be accounted for by host genetic variation. Although no differences were observed in asexual parasite rates and densities among β-globin genotypes, the HbCC genotype was characterized by higher gametocyte rates than the rest of the studied population.

Furthermore, serial infection experiments with blood from CC, AC, AS, and AA donors showed that the protective haemoglobins C (HbC, β6Glu^®^Lys) and S (β6Glu^®^Val) are associated with a twofold *in vivo* (OR 2.17; 95% CI 1.57-3.01; P <0.001) and a fourfold *ex vivo* (OR 4.12; 95% CI 1.90-9.29; P <0.001) increase of parasite transmission from the human host to the *Anopheles* vector.

These findings represent the first demonstration that human genetic variation may also influence the transmission dynamics of an infectious disease. Interestingly, together with previous evidence on the protection against malaria conferred by HbC and HbS, the assembly of the collected parasitological and entomological information suggests that single β globin mutations may confer both a higher resistance to the disease for the host and higher transmissibility for the parasite.
